# Descriptions of immature stages of *Octodonta nipae* (Maulik) (Coleoptera, Chrysomelidae, Cassidinae, Cryptonychini)

**DOI:** 10.3897/zookeys.764.24168

**Published:** 2018-06-05

**Authors:** Ling-fei Peng, Jin-lei Li, You-ming Hou, Xiang Zhang

**Affiliations:** 1 State Key Laboratory of Ecological Pest Control for Fujian and Taiwan Crops; Key Laboratory of Integrated Pest Management for Fujian-Taiwan Crops, Ministry of Agriculture; Fujian Provincial Key Laboratory of Insect Ecology; College of Plant Protection, Fujian Agriculture and Forestry University, Fuzhou, 350002, China

**Keywords:** comparative diagnosis, invasive species, morphology, SEM

## Abstract

*Octodonta nipae* (Maulik, 1921), a hispid that damages several species of palm trees, was introduced accidently into China in 2001. The egg, larva, prepupa and pupa of *O. nipae* are illustrated and described in detail and compared with another invasive species, *Brontispa longissima* (Gestro, 1885); the scanning electron micrographs of the head capsule, antenna, maxilla, labium and lateral scoli are provided, as well as photos of body of all larval instars and pupa. It is the second description of immature stages in the genus *Octodonta* Chapuis.

## Introduction

The genus *Octodonta* Chapuis, 1875 belongs to the tribe Cryptonychini (Chrysomelidae: Cassidinae), which contents eight species distributed in Southeast Asia and one species in Pupa New Guinea. *Octodonta nipae* (Maulik, 1921) is an invasive species which was introduced accidently into Hainan, China in 2001 (Sun et al. 2003), and spread to Fujian in 2007 (Hou and Weng 2010, Hou et al. 2011, Tang et al. 2014a, 2014b, Tang and Hou 2017). *O. nipae* mainly attacks palm tree (Vassiliou et al. 2011, Staines 2017), such as *Nypa fruticans* Wurmb, *Areca catechu* Linn., *Metroxylon sagu* Rottb., *Washingtonia filifera* (Lindl.) H. Wendl. and *Phoenix canariensis* Chabaud, etc. (Maulik 1937, Sun et al. 2003, Li et al. 2014, 2016, Meng et al. 2016). In most cases the beetle feeds on young leaves that do not open or on those that open widely (Steiner 2001, Hou et al. 2014a, 2014b). This causes the young stems to shrink, curl, or die (Zhang 2003). Effective pest control is hard to achieve, because the pest lives within the leaves of the palm and is therefore hard to reach with contact insecticides (Hou and Weng 2010, Hou et al. 2011, Xu et al. 2011, Xi et al. 2013, Feng and Hou 2015).

Many larvae and pupae in the subfamily Cassidinae have been described in detail (Maulik 1938, Gressitt 1960a, 1960b, Ford and Cavey 1985, Borowiec and Świętojańska 2003, Świętojańska et al. 2005, 2006, 2013, 2015, Świętojańska and Borowiec 2007, Świętojańska and Kovac 2007, Świętojańska and Medeiros 2007, Świętojańska and Windsor 2008, Liao et al. 2018), in the genus *Octodonta*, larvae of *O. korthalsiae*, *O. subparallela*, *O. maffinensis* were keyed and described by Gressitt (1960a); and preimaginal stages of *O. depressa* were described by Zaitsev (2006). As the only *Octodonta* species in China, the immature stage of *O. nipae* is still not described in detail, and we found *O. nipae* resembles another invasive species, *Brontispa longissima* (Gestro), especially in the immature stages. Although the morphological and molecular characteristics between *B. longissima* (Gestro) and *O. nipae* were compared (Chen et al. 2015, Zhang et al. 2015) immature stages of these species have not yet been compared. Here we describe the egg, larva and pupa of *O. nipae*, and pupa of *B. longissima*, in order to provide diagnostic characters for the identification of this invasive species which will allow us to differentiate it from *B. longissima* and other species of *Octodonta*.

## Material and methods

Adults of *O. nipae* were initially collected from Fuqing Entry-Exit Inspection and Quarantine Bureau, Fujian Province, China in October, 2007 (Hou and Weng 2010). Subsequently, a colony was established in a laboratory at the College of Plant Protection, Fujian Agriculture and Forestry University. The beetles were reared on center leaves of *Trachycarpus fortunei* (Hook.) H. Wendl. in plastic bottles (diameter 70mm, height 105mm; Jiafeng Horticultural Products Co. Ltd, Fuzhou, China) with moist filter paper to minimize desiccation stress. So far 21 generations have been reared in the laboratory. Larvae of *B. longissima* were collected from Zhangzhou, Fujian Province, China in November 2017. 165 *O. nipae* (eggs, larvae, prepupae, and pupae) and 31 prepupae of *B. longissima* were examined.

The descriptions and illustrations of *O. nipae* egg, larva, prepupa and pupa are based upon laboratory reared individuals. Seta of head description follow Borowiec and Świętojańska (2003), forms of description follow Świętojańska and Kovac (2007) and Świętojańska et al. (2015). Photographs of the specimens were made using a Leica MC170 HD digital camera attached to a Leica M165C microscope. Images were produced using the software Zerene Stacker (Zerene Systems LLC, USA). Drawings were traced from images captured with the camera, then edited with the software Photoshop CS2. For the SEM scanning, the specimens were cleared in xylene for 5 hours, then washed in distilled water. Before dehydration through a graded ethanol series, they were cleaned in an ultrasonic cleaner for one minute, then put in a critical point-drier and held using double-sided adhesive tape and coated with gold in a sputter coater. Examinations were done with a JEOL JSM-6380 LV SEM and HITACHI SU3500.

Measurements are given in millimeters as mean ± SD. Data were analyzed with the SPSS Statistics Version 13.0 for Windows (Table 1).

**Table 1.** Measurements (mean value) of each life stage of *O. nipae* and prepupae of *B. longissima*.

Life stage	Number of specimens	Length of body (mm)	Width of body (mm)
Eggs	37	1.4 ± 0.3	0.5 ± 0.2
1^st^-instar larvae	17	3.0 ± 0.5	1.2 ± 0.3
2^nd^-instar larvae	29	4.5 ± 1.0	1.9 ± 0.5
3^rd^-instar larvae	24	6.8 ± 0.7	2.8 ± 0.3
4^th^-instar larvae	28	7.7 ± 0.4	3.2 ± 0.2
Prepupae	17	7.3 ± 0.1	3.2 ± 0.3
Pupae	13	8.6 ± 0.5	2.9 ± 0.3
Prepupae of *B. longissima*	31	8.99 ± 0.4	2.3 ± 0.2

## Results

### Egg of *O. nipae* (Figs 7–9)

Length 1.4 ± 0.31 mm, width 0.5 ± 0.21 mm. The female adults usually lay eggs in pairs, rarely in triplets or as a single egg. The eggs are surrounded by a sticky secretion and covered with debris and excrement. The newly laid eggs are generally ivory-white, covered with a milky-white soft secretion. After several hours, the eggs turn brown and the secretion becomes sclerotized (Fig. 7). Egg are elongate-oval. The egg chorion is covered with raised networks of ridges (Fig. 8), and the two adjoining polygonal cells share the same ridge (Figs 8–9). The anterior pole of the chorion has smaller cells and taller ridges than the lateral chorion (Fig. 8).

### First instar of *O. nipae* (Figs 1, 10)

**Figures 1–6.**
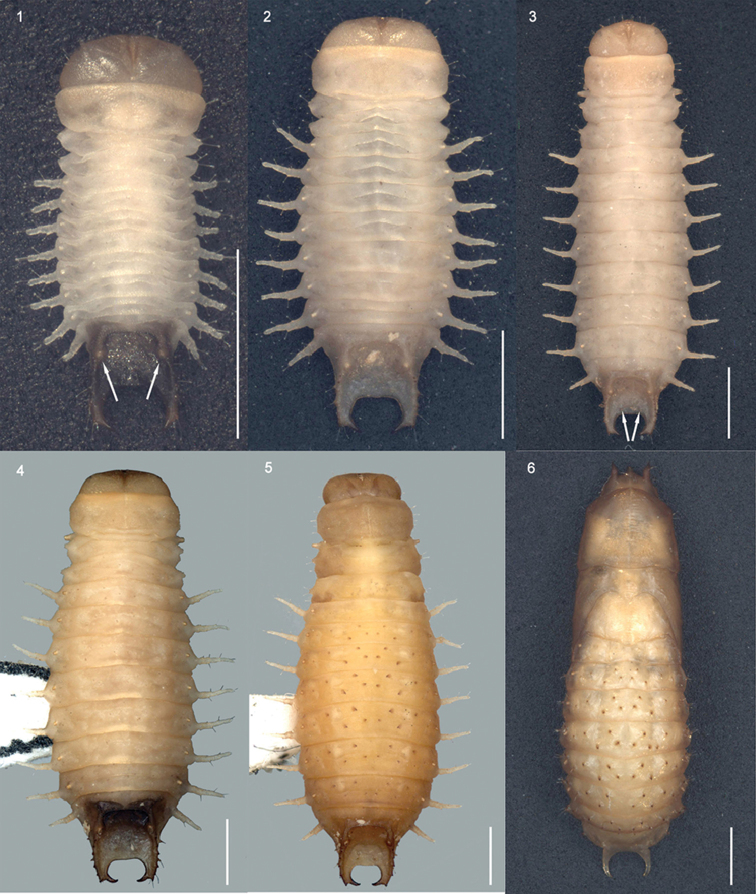
*Octodonta nipae* (Maulik) body dorsal view. **1** first instar (spiracles are indicated) **2** second instar **3** third instar (tubercles are arrowed) **4** fourth instar **5** prepupa **6** pupa. Scale bar: 1mm.

Length 3.0 ± 0.53 mm, width 1.2 ± 0.26 mm. Body flattened dorso-ventrally, lateral margins moderately paralleled, the widest (without lateral scoli) across prothorax (Fig. 1), body surface finely granulate. Head light brown, mandibles dark brown, each lateral side of head with five stemmata and small pigmented spot. Three stemmata distinct round and black placed near antenna, other two partly pigmented (Fig. 10); thorax and abdominal segment I to VII translucent-white, abdominal segment VIII brown or dark brown (Fig. 1).

**Figures 7–15.**
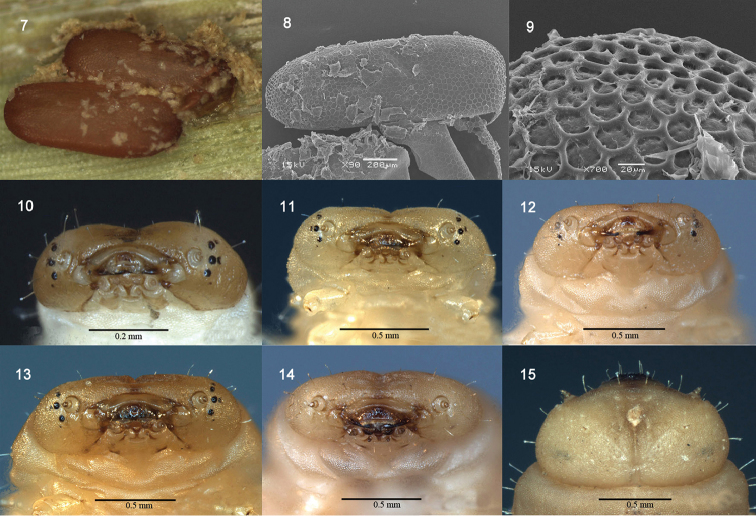
*Octodonta nipae* (Maulik). **7** eggs **8** egg **9** surface of egg **10** head of first larva, front view **11** head of second instar, front view **12** head of third instar, front view **13** head of fourth instar, front view **14** head of prepupa, front view **15** head of prepupa, dorsal view.

Abdomen with eight pairs of lateral scoli on segments I to VIII, and a single pair of supra-anal processes. All lateral scoli not branched, slender and tapering, bearing six club-like setae. Distal segment with supra-anal processes caliper-like hooked, slightly sclerotized, curving slightly inward, each hook with three to four upward directed tiny teeth from base to apex, each teeth with a club-like seta apically.

Spiracles distinctly elevated, round; abdominal spiracles much smaller than mesothoracic spiracle, spiracle of abdominal segment VIII located in inner flank of carina (Fig. 1, arrows indicated).

Prothorax transverse, slightly wider than head, anterior margin convex, surface lightly sclerotized and finely granular; five short setae positioned at lateral margin, three long setae inserted at each ventro-lateral margin. Mesothorax much shorter than prothorax; lateral bulge distinct, bearing two long club-like setae, another long club-like seta on each postero-lateral side (Fig. 1); spiracle elevated, visible in ventral view. Metathorax as long as mesothorax, without spiracle, lateral bulge distinct (Fig. 1).

Head well sclerotized, slightly narrower than prothorax, partially retracted into prothorax (Fig. 1). Epicranial stem absent; median endocarina complete and wide extending between two distinct frontal arms, from close to the center of the posterior margin up to the position of the antenna; fronto-clypeal suture present, clypeo-labral suture present. Clypeus distinct, much wider than long. Antenna very short, three-segmented set in membranous ring, anterolaterally directed; segment I and II ring-like, one large sensorial appendage inserted ventrally on segment II, below segment III; segment III parallel-sided and as long as the sensorial appendage (Fig. 10). Five stemmata positioned behind the antenna, four of them in a row, another one positioned further back, pigmented spot placed below row of four distinctly marked stemmata (Fig. 10). Labrum sclerotized, wider than long, six setae positioned dorsally, anterior part with thick stout curve spines.

### Second to fourth instar of *O. nipae* (Figs 2–4, 11–13)

Body length 4.5–7.7 mm, width 1.9–3.2 mm (Table 1). Abdomen wider than head and thorax. Head capsule much wider than long, anterior margin convex and evenly rounded laterally, finely granular. Supra-anal processes strongly carinate and sclerotized, curving slightly inward, each dorsal carina with four to six upward directed large teeth from base to apex, lateral carina bearing with two to four large teeth; two setae positioned on tiny tubercle near inner margin of processes (Fig. 3, arrow indicated).

### Prepupa of *O. nipae* (Figs 5, 14, 15, 16–45)

Length 8.0 ± 0.42 mm, width 3.4 ± 0.32 mm. Head light brown, mandibles black, labrum dark brown, stemmata concolorous with surrounding area, no pigment (Fig. 14); thorax and abdominal segment I to VII light brown, anterior margin and lateral scoli of abdominal segment VIII brown, last segment brown or dark brown (Fig. 5); each abdominal tergum II to VII with ten dark brown sclerotized spines (Fig. 5).

Body flattened dorso-ventrally, elongate-oval, widest across abdominal segment V. Abdomen with eight pairs of lateral scoli and a pair of short supra-anal processes (Fig. 5). Lateral scoli slender and tapering, finely denticulate (Figs 5, 33) and bearing six long club-like setae (Fig. 25). Supra-anal processes caliper-like hooked, strongly carinate and sclerotized, each dorsal carina with five to six upward directed large teeth from base to apex, lateral carina bearing three to four large laterally directed tubercles with setae at apex, two setae positioned on tiny tubercle near inner margin of processes (Fig. 34, arrow indicated).

Setae of head club-like, blunt apically or pointed, club-like setae more or less of the same length with scoli setae, but some setae of head very short (Figs 22, 23, 27, 28). Setae of tergites short and pointed (Figs 35–38). Setae of legs club-like or long pointed (Fig. 43).

Dorsal side of prothorax with five pairs of tiny pointed setae (Figs 35, 44), four pairs arranged in row near posterior margin, one pair near middle of tergite; three pairs of blunt apical setae near anterior margin; five pairs of club-like setae along lateral margin. Dorsal side of mesothorax with five pairs of tiny pointed setae arranged in row along posterior margin (Figs 36, 44); three blunt apical setae near bulge and spiracle; three long club-like setae along lateral margin, two of them on the bulge and one behind the bulge. Dorsal side of metathorax with nine pairs of tiny pointed setae (Figs 37, 38, 44), two pairs positioned antero-quarterly of anterior margin, four pairs arranged in row along middle of tergite, three pairs of tiny pointed setae and one blunt apical setae arranged in row along postero-lateral margin; four pairs of club-like setae along lateral margin. Terga II to VII with ten setae placed on tubercles (Figs 5, 44): one pair near spiracle, two pairs near anterior margin, one pair near posterior margin, and another pair positioned centrally.

Nine pairs of spiracles (Fig. 5): one pair on the mesothorax and eight on abdomen. Mesothoracic spiracle tubular and distinct (Figs 5, 29), positioned laterally just behind prothorax; abdominal spiracles small, inner wall of spiracles finely granulate, entrance opened (Figs 30, 31). Spiracle of last segment abdomen round, larger than other abdominal spiracle, located in inner flank of dorsal carina (Figs 32, 34).

Head (Figs 14, 15, 17, 44) well sclerotized, shallowly retracted into prothorax, distinctly wider than long, but narrower than prothorax, lateral margin strongly rounded; dorsal surface finely granular, median endocarina well developed, widening and deepening from posterior margin to clypeal posterior margin, frontal arms extending from close to the center of the posterior margin up to the position of the antenna.

Stemma (Figs 14, 17). Position of stemmata similar with younger instars. All the stemmata concolorous with surrounding area, pale brown (Fig. 14).

Head with numerous setae, distribution of setae as shown in Figure 40.

**Figures 16–21.**
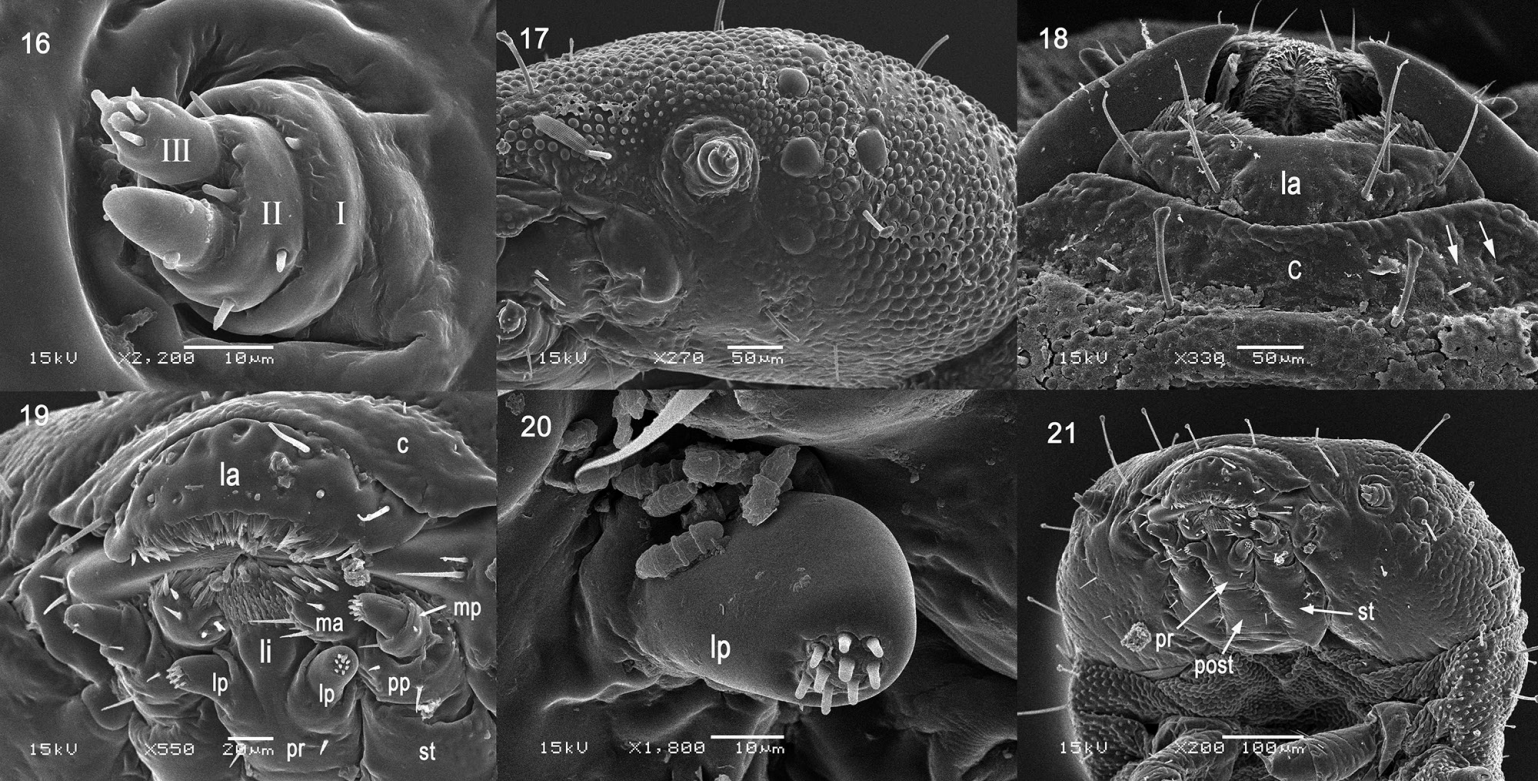
Prepupa of *Octodonta nipae* (Maulik). **16** antenna **17** head, latero-front view **18** clypeus and labrum **19** mouthpart, ventral view **20** labial palp **21** head, ventral view. Abbreviations: c – clypeus; la – labrum; lp – labial palp; li – ligula; ma – mala; mp - maxillary palp; post – postmentum; pp – palpifer; pr – prementum; st - stipes.

Antenna (Figs 16, 24, 41). Antenna very short, three-segmented, anterolaterally directed set in membranous ring; segment I ring-like, with one tiny seta placed laterally (Figs 16, 41); segment II slightly wider than long, with one large conical sensory appendix apically, three setae laterally (Fig. 24) and one small peg-like sensillum placed between sensory appendage and antennal segment III; segment III as long as segment II, apical portion with six peg-like sensilla (Figs 16, 41).

Labrum bent down (Figs 18, 19), connected with the anterior clypeal margin, narrower than clypeus. Anterior margin with tufted setae, outer surface of labrum with six long pointed setae (Fig. 18).

Clypeus narrow and wide (Fig. 18), moderately sclerotized, arched in front view, anterior and posterior margin parallel. One blunt apical seta and one tiny pointed seta positioned latero-posterior (Fig. 18, arrows pointed).

Mandible heavily sclerotized (Figs 18, 42), triangular, short and compact, with three apical teeth; inner side of mandible sharp, dorsal side of mandible convex, dorsolateral side with one long and one short pointed seta (Figs 19, 42).

Maxilla with stipes long (Fig. 19), bearing two lateral setae. Mala larger than maxillary palp (Fig. 19), directing buccal cavity, with a large cluster of setae apically, seven long pointed setae below the cluster setae. Palpifer short (Fig. 19), with two setae, medial seta distinctly longer than lateral one. Maxillary palp short (Fig. 19), segment I cylindrical, with two setae, segment II conical, longer than segment I, three setae positioned flank, apical area with 11 sensilla. Labial palp one segmented (Figs 19, 20), finger-like, apex with a group of nine peg-like sensilla. Hypopharynx apex covered with numerous spines (Fig. 19). Submentum and mentum fused with basal portion of maxilla, one pair of setae near lateral margin of postmentum (Fig. 21). Prementum narrow with two short pointed setae (Fig. 21). Postmentum with two long pointed setae (Fig. 21).

Leg three-segmented (Figs 29, 43); coxa much wider than long, with setae arranged in three rows: first with two setae, second with three setae, third with two setae and one club-like seta; femur 1.6 times longer than wide, with seven long setae, two club-like setae, and one short seta close to the base, as show in figure 43; tibiotarsus stout, apically with one heavily sclerotized and curved single claw, armed apically six long setae around claw, and one club-like seta at middle.

**Figures 22–28.**
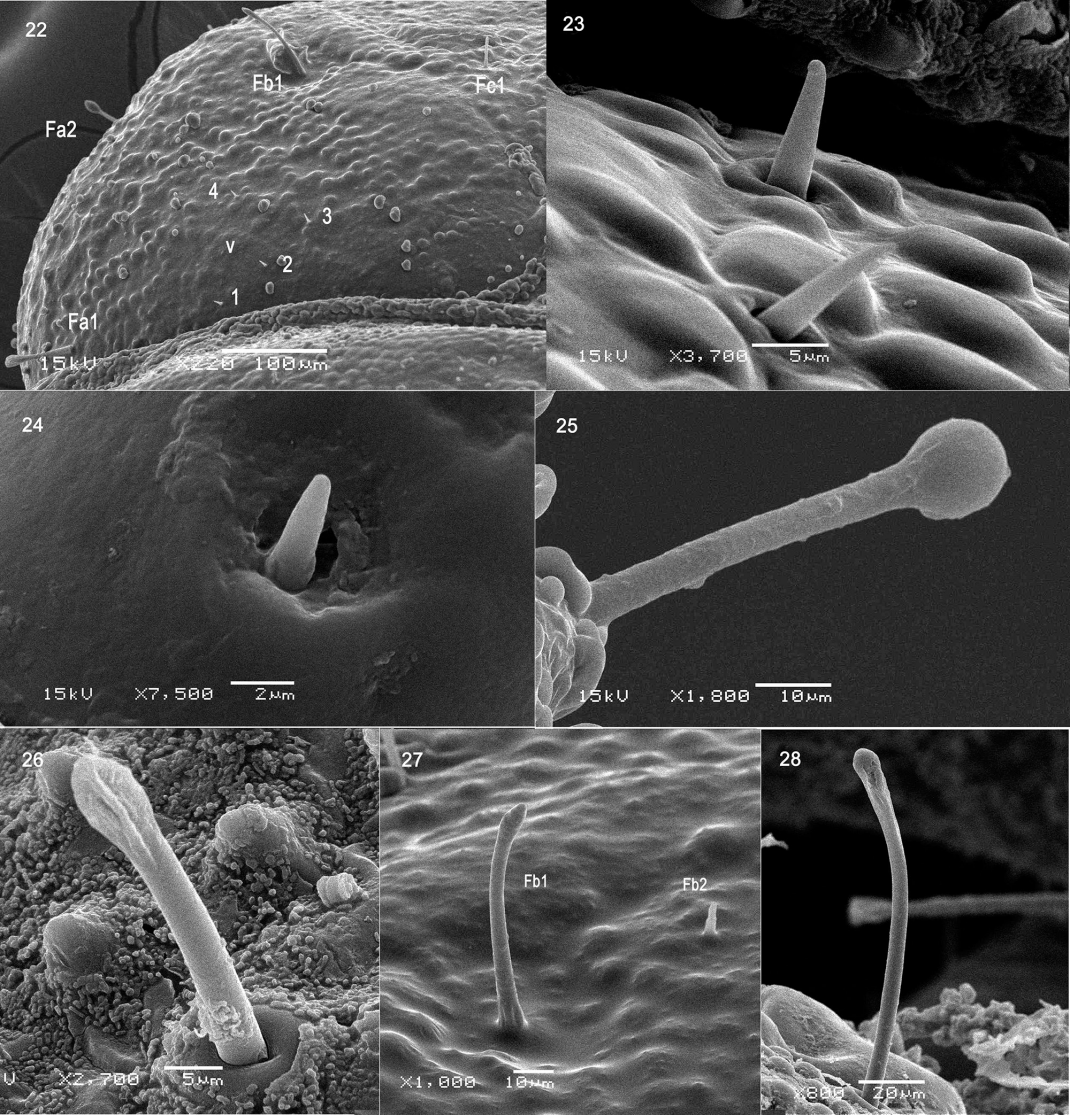
Prepupa of *Octodonta nipae* (Maulik). **22** part of head, dorsal view **23** vertical setae of head **24** setae of antenna **25** seta of scolus **26** seta of abdomen **27** seta of head (Fb1, Fb2) **28** seta of head (Fc2). Abbreviations: Fa/b/c – frontal setae, row a/b/c; v – vertical setae.

**Figures 29–32.**
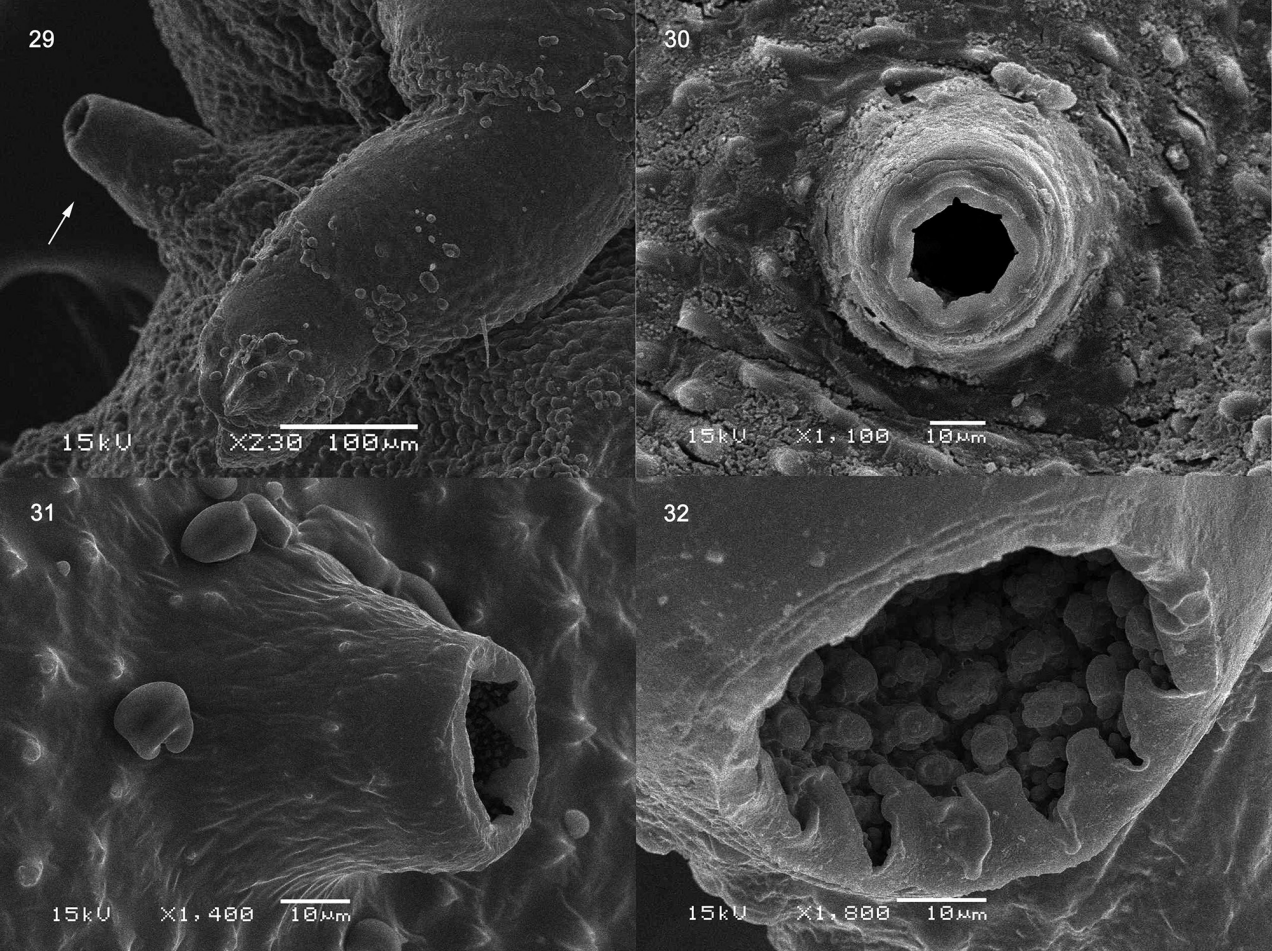
Prepupa of *Octodonta nipae* (Maulik). **29** proleg and spiracle of mesothorax **30** spiracle of abdomen **31, 32** spiracle of last abdominal segment.

**Figures 33–39.**
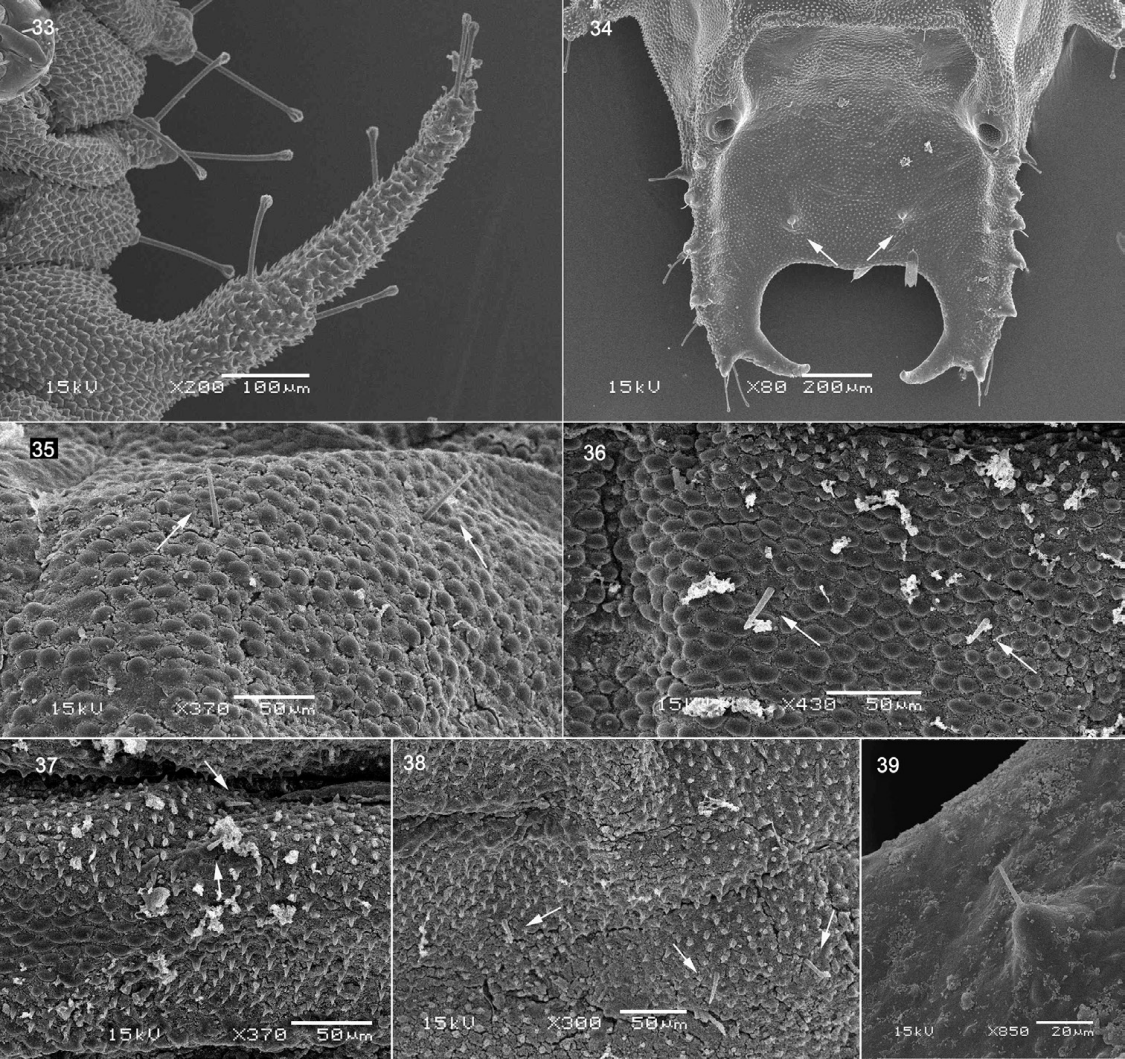
Prepupa of *Octodonta nipae* (Maulik). **33** scolus; **34** supra-anal process (showing the setae) **35** setae of prothorax **36** setae of mesothorax **37**, **38** setae of metathorax **39** seta of supra-anal process.

**Figures 40–45.**
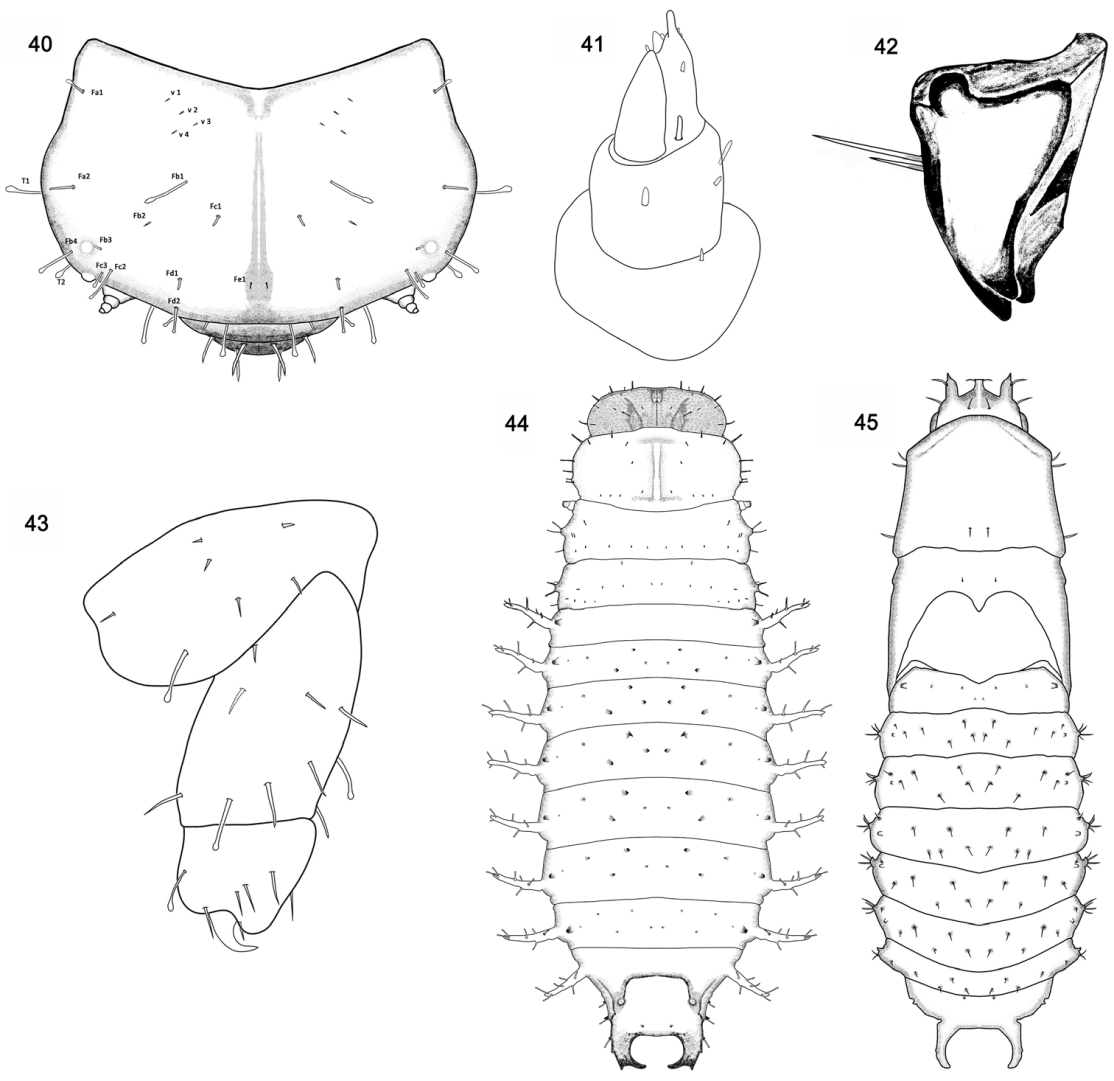
*Octodonta nipae* (Maulik). **40** frontal side of head **41** antenna **42** mandible **43** leg **44** prepupa, dorsal view **45** pupa, dorsal view.

### Pupa of *O. nipae* (Figs 6, 45–47)

**Figures 46–48.**
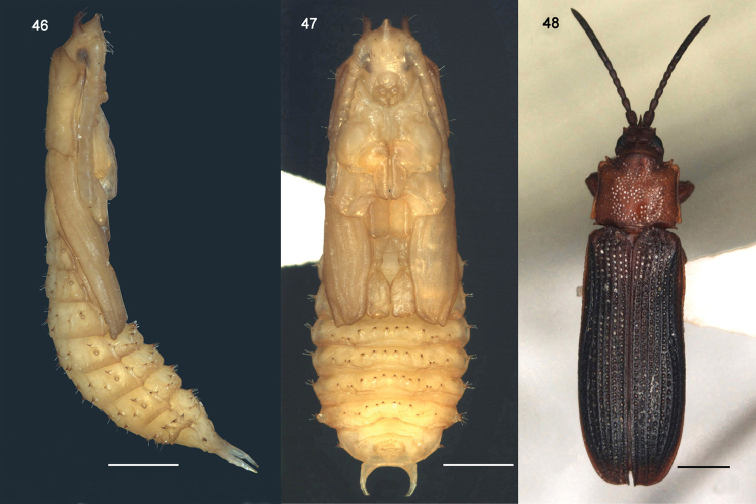
*Octodonta nipae* (Maulik). **46** pupa, lateral view **47** pupa, ventral view **48** adult, dorsal view.

Length 8.6 ± 0.51 mm, width 3.6 ± 0.34 mm. Body long oval, exarate, flattened dorso-ventrally; body straight from apex of head to abdominal segment III, bent ventrally at abdominal segment IV (Figs 46, 47). Color light brown, eyes dimly black (Fig. 47).

Head with three stout processes in dorsal view, one central process with apex truncated and bearing two lateral setae (Fig. 45); two lateral processes positioned laterally, with apex acute and bent ventrally, one lateral setae inserted near apex (Fig. 45). In ventral view, eyes, antennae, labrum, mandibles, and maxilla discernible (Fig. 47).

Pronotum large, shield like, anterior margin convex, lateral margin truncate with three setae, two anterior and one posterior, posterior margin slightly sinuate; one pair of setae positioned centrally on posterior disc (Fig. 45). Mesonotum with lateral margin slightly sinuate, elytral theca closely appressed with wing theca, curved ventrally around body, extending to abdominal segment IV (Figs 6, 45–47). Leg theca stout, closely appressed with body, metapedes not extending beyond apex of elytral theca (Fig. 47).

Abdominal segment I to VIII visible in dorsal view, each segment with one pair of spiracles, and a large number of hooked spines and setae (Figs 6, 45). Tergum of first segment with six small spines (Fig. 45). Tergum II to VII marked with eight pairs of hooked spines, five pairs located between two spiracles, each spine bearing one seta (Fig. 45); one pair of spines near spiracle which bears one seta (Fig. 45); two pairs of spines positioned on the lateral margin of tergum, one directed dorsally and one directed ventrally, each of them bearing two setae (Fig. 45). Tergum VIII with supra-anal processes long hooked (Fig. 45), spiracles of tergum VIII in same locality as in larva. Sternum IV to VIII visible, sternum IV to VII bearing 12 hooked spines; sternum VIII without spine, anus positioned centrally, transverse and oval shape, last abdominal segment slim and soft (Figs 45–47).

### Prepupa of *B. longissima* (Figs 49–60)

Body elongate, flattened dorso-ventrally. Abdomen with eight pairs of lateral scoli and a pair of short supra-anal processes (Fig. 59). Lateral scoli short and tapering, finely denticulate and bearing four long club-like setae (Fig. 58). Supra-anal processes caliper-like hooked, strongly carinate and sclerotized, each dorsal carina with five to seven upward directed large teeth from base to apex, lateral carina bearing three to four large laterally directed tubercles with setae at apex (Fig. 59).

Most setae of head hair-like or club-like, blunt apically or pointed, but some setae of head very short (Fig. 57). Setae of tergites short or long, pointed, blunt or club-like (Fig. 52). Setae of legs club-like or long pointed, two setae positioned on tiny tubercle at inner margin of processes (Fig. 59)

Dorsal side of prothorax with four pairs of pointed setae arranged in row near posterior margin, one pair near middle of tergite; four pairs of blunt apical setae near anterior margin; six pairs of club-like setae along lateral margin (Fig. 50). Dorsal side of mesothorax with four pairs of pointed setae arranged in row posterolaterally; three pairs of pointed setae arranged in row near middle of tergite; two pairs of tiny pointed setae near anterior margin; one pair of blunt apical setae positioned anterolaterally; three long club-like setae along lateral margin, two of them on the bulge and one behind the bulge (Fig. 50). Dorsal side of metathorax with seven pairs of tiny pointed setae (Fig. 50), four pairs arranged in row posterolaterally; three pairs arranged in row along middle of tergite; one pair of blunt apical setae positioned near anterolaterally; three pairs club-like setae and one pointed seta along lateral margin (Fig. 50).

**Figures 49–51.**
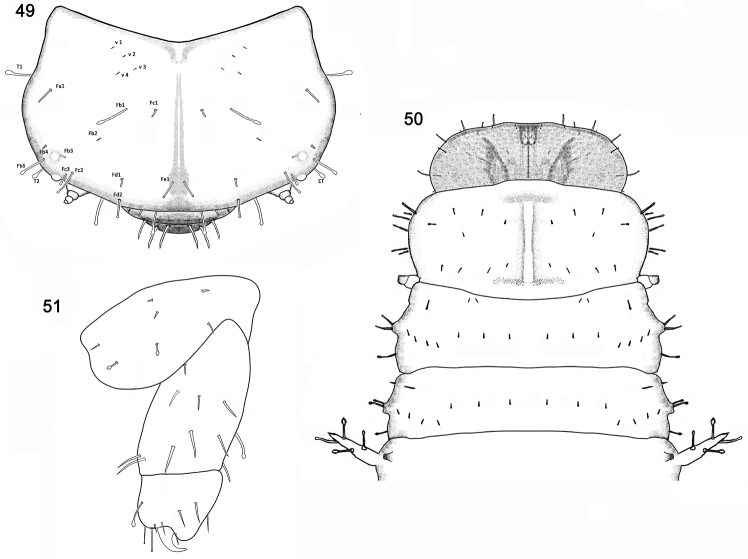
*Brontispa longissima* (Gestro). **49** frontal side of head **50** head, thorax and **1**^st^ segment of abdomen, dorsal view **51** leg.

Nine pairs of spiracles: one pair on the mesothorax and eight on abdomen. Mesothoracic spiracle tubular and distinct (Fig. 50) laterally positioned between pro- and mesothorax; abdominal spiracles small. Spiracle of last segment abdomen round, larger than other abdominal spiracle, located in inner flank of dorsal carina (Fig. 59).

**Figures 52–60.**
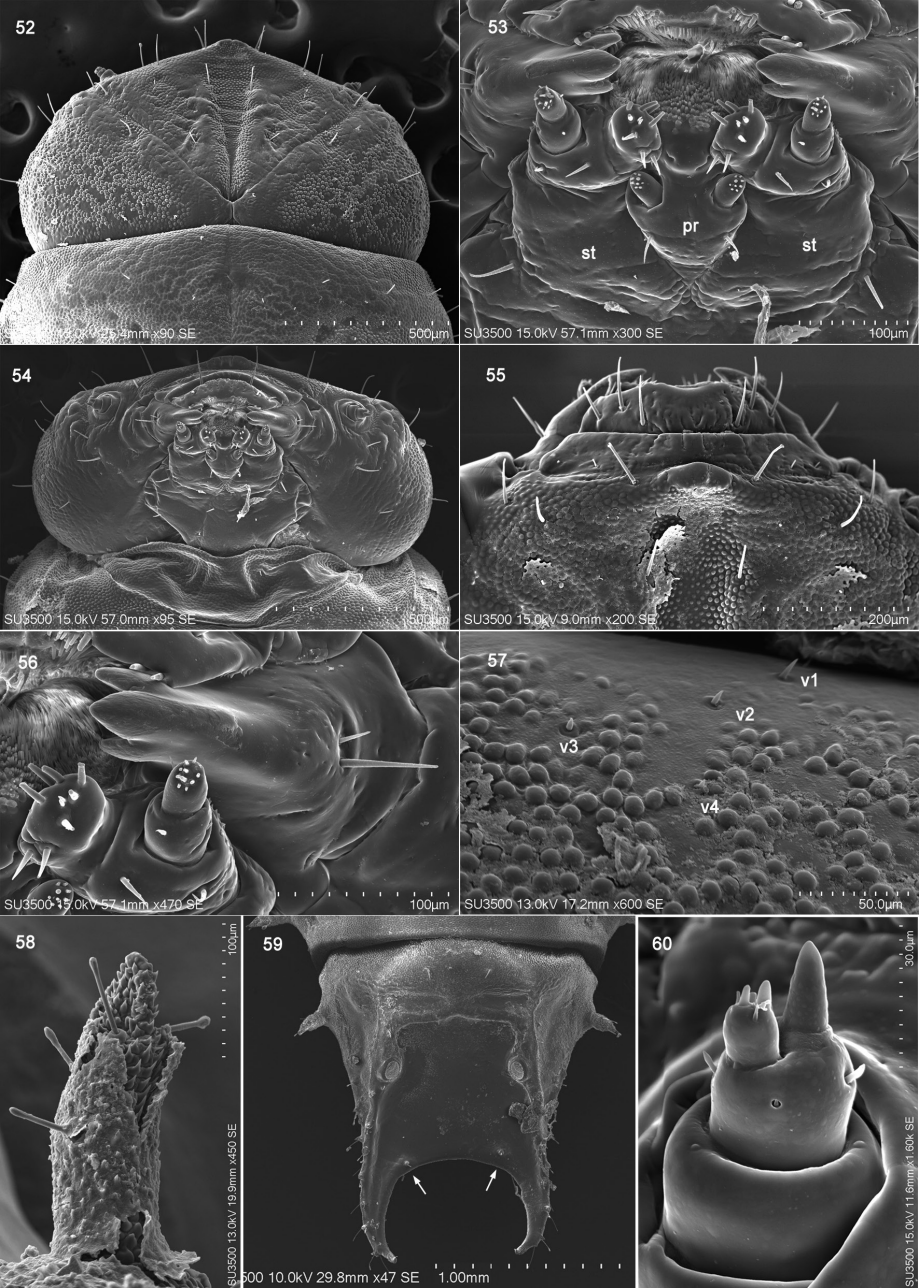
*Brontispa longissima* (Gestro). **52** head, dorsal view **53** mouthpart, ventral view **54** head, ventral view **55** clypeus and labrum **56** mandible **57** vertical setae of head **58** scolus **59** supra-anal process **60** antenna. Abbreviations: pr – prementum; st – stipes; v – vertical setae.

Head (Figs 49, 52) distinctly wider than long, but narrower than prothorax; dorsal surface finely granular, median endocarina well developed, widening and deepening from posterior margin to clypeal posterior margin, frontal arms extending from close to the center of the posterior margin up to the position of the antenna (Fig. 51).

Head with numerous setae (Figs 49, 51), distribution of setae as shown in Figure 49.

Labrum bent down (Figs 54, 55), connected with the anterior clypeal margin, almost as wide as clypeus. Anterior margin with tufted setae (Fig. 53), outer surface of labrum with six long pointed setae (Fig. 55).

Clypeus narrow (Fig. 55), moderately sclerotized, arched in front view, anterior and posterior margin parallel. Two pairs of tiny pointed seta positioned laterally (Fig. 55).

Mandible heavily sclerotized (Figs 53, 56), short and compact, with three apical teeth; inner side of mandible sharp, dorsal side of mandible convex, dorsolateral side with one long and one short pointed setae (Fig. 56).

Maxilla with stipes long (Figs 53, 54), bearing two lateral setae. Mala larger than maxillary palp (Fig. 53), directing buccal cavity, with eight long pointed setae and two short pointed setae. Palpifer short (Fig. 53), with two pointed setae. Maxillary palp short (Fig. 53), segment I cylindrical, with two setae, segment II conical, as long as segment I, one setae positioned flank, apical area with 11 sensilla. Labial palp one segmented, short (Fig. 53), apex with a group of nine peg-like sensilla. Hypopharynx apex covered with numerous spines (Fig. 53). Submentum and mentum fused with basal portion of maxilla, one pair of setae near lateral margin of postmentum (Figs 53, 54). Prementum triangular with two short pointed setae (Fig. 54). Postmentum triangular with two long pointed setae (Fig. 54).

Leg three-segmented (Fig. 51); coxa wider than long, with setae arranged in three rows: first with two pointed setae, second with two pointed setae and one club-like seta, third with one pointed seta and one club-like seta; femur longer than wide, with 11 long pointed setae, one club-like setae, as show in figure 51; tibiotarsus stout, apically with one heavily sclerotized and curved single claw, armed apically nine long setae around claw.

## Discussion

The first-instar larva of *Octodonta nipae* with the combined length of head and thorax make up more than one-third of body length, while in the other larval instars head and thorax account for no more than one-third of body length. The mesothoracic spiracles are invisible in dorsal view in the first and second instar larvae, but the third and fourth instar larvae and prepupa have remarkably long tubular mesothoracic spiracles, which are dorsally visible. The first-instar larva with the supra-anal processes slightly sclerotized and only bearing one to three small teeth; older larvae have a strongly carinate and sclerotized supra-anal processes and more teeth; the last abdominal segment of the pupa is very soft and narrower than in the prepupa (Fig. 1), wrapped closely with crimping exuvium. The third and fourth instar larvae and prepupa have two tubercles which are positioned at the inner margin of the last abdominal segment (Figs 3–5); the first and second instar larvae lack tubercles and only have two tiny setae, which are almost invisible (Figs 1, 2).

The egg and larva of *Brontispa longissima* were described in detail by Maulik (1938), in his description we found several diagnostic characters distinguishing the immatures of *O. nipae* and *B. longissima*. Eggs of both species are elongate and ellipsoidal; similar in size and sculpture. The first instar larva of *B. longissima* has all lateral scoli bearing two setae while in *O. nipae* lateral scoli have six club-like setae; the last abdominal segment of *B. longissima* is caliper-like hooked and has a series of five or six hairs along dorsal margin, but in *O. nipae* the dorsal margin has three to four club-like setae.

According to our observation, the mature larva of *O. nipae* resembles the larva of *B. longissima*, but there are many differences:

1) The body length of *O. nipae* (7.32 ± 0.06 mm) shorter than *B. longissima* (8.99 ± 0.38 mm).

2) The setae distribution of head is different as shown in pictures, row of Fa of *B. longissima* is only with one seta Fa1, but row of Fa of *O. nipae* with two setae; row of Fb of *B. longissima* is with five setae, but row of Fb of *O. nipae* with four setae.

3) Scoli of *O. nipae* are conical and slender, bearing six club-like setae; but *B. longissima* has the scoli which bearing four long club-like setae, and are shorter than the scoli of *O. nipae*;

4) The setae distribution of thorax is different (Figs 44, 50).

5) The setae distribution of leg is different (Figs 43, 51).

6) The supra-anal processes all caliper-like hooked, *O. nipae* with two small pointed setae positioned near inner margin of processes (Fig. 34, arrows indicated), but these two setae of *B. longissima* are closer to the inner margin (Fig. 59, arrows indicated). We also found some different from the description of Maulik (1938), in his description the mandibles of *B. longissima* are with two teeth, but we found the mandible of *B. longissima* with three teeth (Fig. 56) as *O. nipae*; Maulik said “*B. longissima* with the spiracle of mesothorax is not visible dorsally”, but according to our observation the spiracles of mesothorax are visible dorsally (Fig. 50).

Gressitt (1960a) keyed and simply described larvae of *O. korthalsiae*, *O. subparallela*, and *O. maffinensis*. Compared with his descriptions, *O. nipae* resembles *O. korthalsiae*, they all have long and slender scoli, last abdominal segment strongly arched and toothed; but the scoli of *O. korthalsiae* are as long as length of head, in *O. nipae* the scoli are 1.8 times as long as head.

The larva of *O. depressa* was described and figured by Zaitsev (2006), the mandible is triangular, with one tooth and a sharp inner edge, outer side has one seta; antennae are three-segmented, segment II has one small triangular sensillum apically and segment III long and slender; scoli have seven setae; last abdominal segment with each dorsal carina have five sharp teeth from base to apex, each lateral carina only have one laterally directed tooth near apex. The mandible of *O. nipae* larva has three teeth at inner side, outer side has two long setae; antennae are three-segmented, but segment II has a large conical sensory appendix apically and one small peg-like sensillum, segment III is conical and apically bearing with six peg-like sensilla; scoli have six setae; last abdominal segment with each dorsal carina have five to six teeth, and each lateral carina have three to four teeth laterally directed.
